# Eosinophil protein X and childhood asthma: A systematic review and meta‐analysis

**DOI:** 10.1002/iid3.104

**Published:** 2016-04-01

**Authors:** Hillary Klonoff‐Cohen, Mounika Polavarapu

**Affiliations:** ^1^ Department of Kinesiology and Community HealthUniversity of Illinois Urbana‐ChampaignHuff Hall Room 2021 A, 1206 S. Fourth St.ChampaignIL61820

**Keywords:** Childhood asthma, cut points, inflammatory markers, meta‐analysis, review article, serum EPX, urine EPX

## Abstract

Background: There are no reference guidelines for health care providers regarding appropriate use and interpretation of urine eosinophil protein X (u‐EPX) in clinical practice. Currently, there are no clear‐cut clinical or laboratory parameters to diagnose asthma in young children. Objective: In this study, we (1) systematically reviewed and qualitatively appraised the epidemiological evidence to determine diagnostic u‐EPX cut points for pediatric asthma, and (2) performed a meta‐analysis to provide u‐EPX estimates for diagnosing pediatric asthma. Methods: Research articles in literature were identified from PubMed/Medline and Web of Science databases from 1966 to August 2015. Children <18 years of age were included. Both serum and urine EPX were included. Twenty‐seven studies met the inclusion criteria for the systematic review and nine studies for the meta‐analysis. Details regarding EPX analyses, treatment efficacy, and outcomes were assessed. For meta‐analyses, effect estimates were abstracted using standardized means. Results: Over 70% of studies found a significant relationship between u‐EPX and childhood asthma. There was 1.94 times higher standardized means of u‐EPX among acute asthmatics compared to healthy controls (confidence interval [CI]: 1.67–2.22). Similarly, the difference in standardized means between asymptomatic asthmatics and healthy controls was 1.58 times higher (CI: 1.27–1.88). Conclusions and Clinical Relevance: Despite differences in sample sizes, EPX processing and measurement, and ages of children, a consistent trend of higher EPX levels with childhood asthma was revealed.

## Background

### Childhood asthma

Asthma is a clinical syndrome characterized by airway inflammation and hyper‐responsiveness, often triggered by respiratory infections, inhaled allergens, cold, wind, or dust 1. It is the most common chronic condition in children 2. In the United States, more than 10 million children under age 18 have been diagnosed with asthma 3. This disease is the third‐ranking cause of hospitalizations in children 4, and accounts for approximately 175,000 annual hospitalizations 5. In the United States, the national annual healthcare cost for pediatric asthma is approximately $3 billion 6.

Problems with managing childhood asthma occur because of non‐specific symptoms, as well as the inability to obtain reliable pulmonary function test (PFT) results, particularly in children below the age of 6 years 7.

### Inflammatory markers

At this time, no single outcome provides a reliable measure for diagnosing asthma status. This is an area in which markers of inflammation may have a great impact, but their value in routine care has yet to be established. Currently, inflammatory markers do not appear to have a clinical role outside of research.

An ideal marker for identifying childhood asthma should (1) reflect spontaneous changes in disease activity, (2) reflect improvements due to therapy, (3) have high sensitivity and specificity, (4) have prognostic significance, (5) be reproducible in steady state, (6) be fast, easy to obtain, and non‐invasive, and (7) be inexpensive 8. Several inflammatory markers for asthma have been tested in the past decade, including serum ECP, exhaled nitric oxide, and C reactive protein.

An inflammatory marker that is easy to collect in young children is vitally important. Of the four basic eosinophil granule proteins (i.e., major basic protein, eosinophil cationic protein, eosinophil protein X [EPX], and eosinophil peroxidase), EPX, also known as eosinophil‐derived neurotoxin (EDN), is the only marker that can be accurately measured in urine 9. However, the precise role of urinary eosinophil protein X (u‐EPX) in the diagnosis of asthmatic disease remains controversial 10.

### Eosinophil protein X

EPX may be the most valuable biomarker of eosinophil activation in childhood asthma 11 because it is sensitive, non‐invasive, and easily measured in urine. Urinary eosinophil protein X (u‐EPX) correlates inversely with nocturnal peak expiratory flow rates (PEFRs) and 1‐second forced expiratory volume, reflecting its role as a marker in asthma activity 11. u‐EPX does not undergo significant in vivo degradation and is not influenced by perennial allergy and polysensitization 12 and can differentiate symptomatic from asymptomatic patients.

There is a much higher release of EPX than serum eosinophil cationic protein (s‐ECP) in asthmatics, perhaps, indicating that eosinophils selectively release their granule proteins depending upon the type of stimulus to which the cells are exposed 13. Thus, the determination of EPX in urine gives the same, if not better, information on disease activity and impact of treatment on inflammation as s‐ECP.

Currently, normal reference ranges for u‐EPX in children with asthma and healthy controls are lacking. Hence, the objective of this meta‐analysis is to determine whether u‐ EPX is a valuable marker in diagnosing childhood asthma. Additionally, preliminary cut points will be proposed for EPX levels in asthmatics and healthy children. Both of these aims will be useful in guiding clinical practice for pediatric asthma.

## Methods

### Data sources, search strategy, and study selection

**Figure 1:**
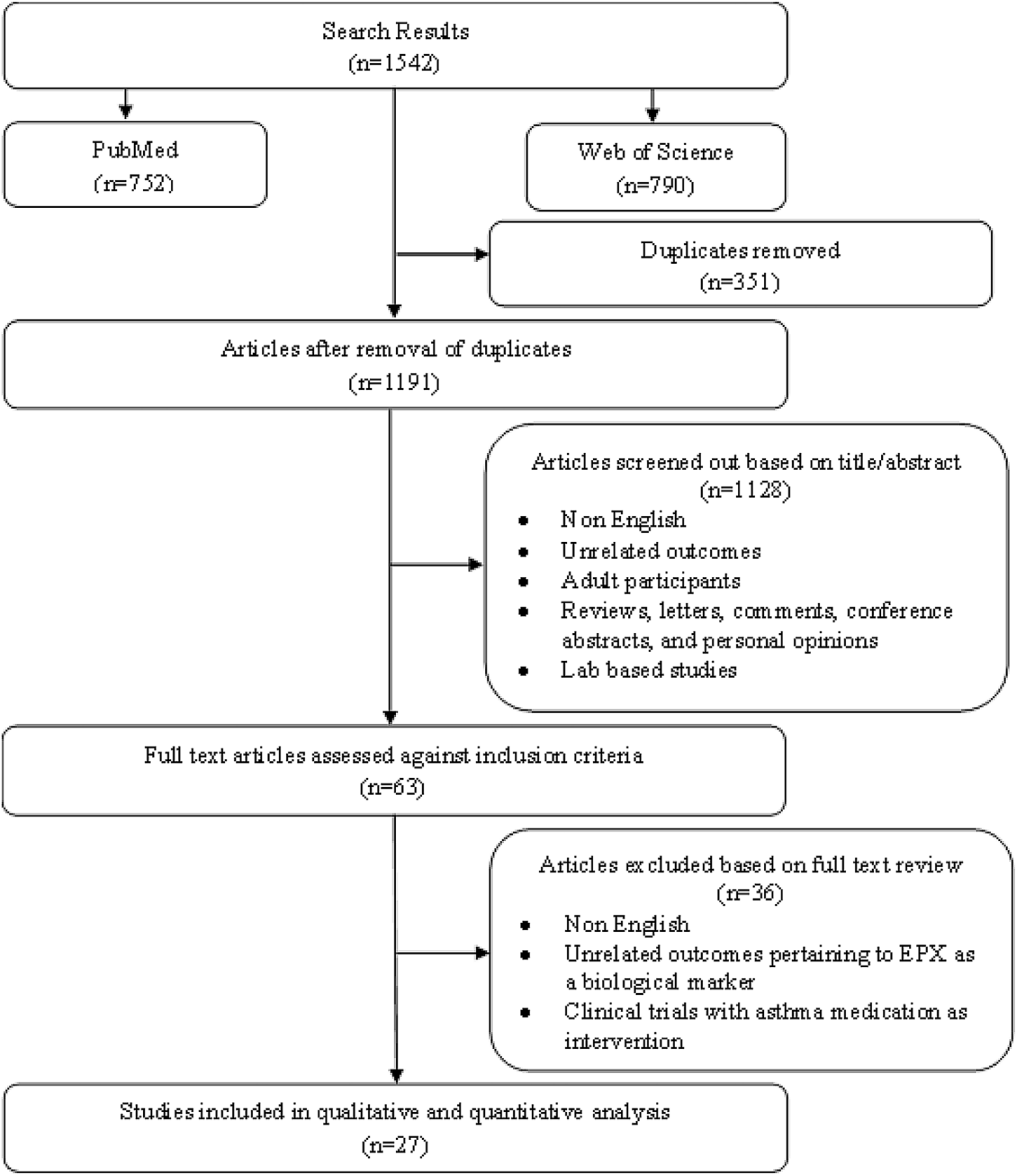
Flow chart of literature search and selection criteria.

A systematic literature search of PubMed/Medline and Web of Science core collection databases were performed for studies published until August 2015, using the search terms, *childhood asthma, pediatric asthma, serum EPX, urine EPX, Eosinophil protein X, Eosinophil‐derived neurotoxin, EDN, and inflammatory markers*. The search strategies are reported in detail in the flowchart in Figure 1.

Using the title and abstract, the literature search was reviewed independently in quadruplicate (H. Klonoff‐Cohen, J. Lee, O. Refugio, M. Polvarapu) and screened against the study selection criteria to identify potentially relevant studies for full review. After the removal of duplicates, a total of 1191 articles were found from electronic databases, and from reference mining. Searches of biographies were also conducted to ascertain additional studies. Relevant studies were selected, assessed and data were extracted independently by four people (H. Klonoff‐Cohen, J. Lee, O. Refugio, and M. Polvarapu). Disagreement was resolved by discussion and mutual agreement among the reviewers. Of the 1191 articles identified, 1128 articles were excluded based on the article's title and abstract (not relevant), and 63 articles were retrieved for full text review. After complete assessment, only 27 articles met the established inclusion criteria. Authors of these 27 articles were contacted at least three times to obtain missing information.

### Eligibility criteria

The following inclusion criteria were used for the systematic review: (1) Peer reviewed articles written in English; (2) cohort studies, cross‐sectional studies, and case–control studies; (3) asthma as the index disease; (4) study sample: Children up to 17 years of age; (5) urinary and/or serum EPX used as a biological marker; (6) assessment of EPX in asthmatics and healthy control children. Review studies were excluded. Additionally, one randomized clinical trial was excluded because of the design. In this study, asthma patients were randomly assigned deliberate doses of montelukast in order to understand the variation in eosinophilic protein markers with medication. In all other studies, patients received treatments based on physician prescribed medications rather than being randomly allocated to pre‐defined treatment groups. Hence, a total of 27 studies that complied with the pre‐stated inclusion criteria were identified.

Among the 27 selected studies, for quantitative analysis (meta‐analysis), the criteria used for inclusion were (1) presence of control group in the study consisting of healthy non‐asthmatic children; (2) study group consisting of acute asthmatics OR asymptomatic asthmatics during the collection of urine sample; (3) reported values of u‐EPX among asthmatics and healthy controls with measures of center and spread (mean/median; standard deviation/interquartile range); and (4) for a valid comparison, all the values of u‐EPX reported in units μg/mmol Creatinine.

### Data extraction

The results from studies that met the inclusion criteria and contained the outcomes of interest were included in the meta‐analyses. All data were double entered and triple checked.

Summary outcome measures consisting of urinary EPX levels for childhood asthmatics/non‐asthmatics, symptomatic/asymptomatic asthmatics, those on medication/without medications, atopic asthmatics/non‐atopic asthmatics, as well as children experiencing acute exacerbations were extracted from studies and tabulated. Following data were extracted from the studies identified for this manuscript: author, publication year, study hypothesis, study design, sample size and characteristics of study and comparison groups, EPX measures, conclusion, and disadvantages of the studies. We also assessed the methodology used to measure u‐EPX levels with the data, including test kit, detection limit/sensitivity of the kit, time and rate of centrifuging, freezer temperature, duration before freezing the sample, and any additional unique information provided pertaining to the process.

### Assessing the quality of studies

The quality of each of study included in the quantitative analysis (meta‐analysis) was assessed using the dichotomous criteria (Table 1) which was designed based on the “Quality Assessment Tool for observational cohort and cross‐sectional studies,” “QUADAS 2,” and “Newcastle‐Ottawa scale.”

**Table 1:** Quality assessment criteria for studies included in meta‐analysis

Criteria	Yes	No
Research question		
Was the research question or objective in the paper clearly stated?	✓	X
Study population		
Was the study population clearly specified and defined?	✓	X
Selection bias		
Are the individuals selected to participate in the study likely to be representative of the target population?	X	✓
Inclusion and exclusion criteria		
Were inclusion and exclusion criteria for being in the study pre‐specified and applied uniformly to all the participants?	✓	X
Measurement of exposure		
Were the exposure measures clearly defined, valid, reliable, and implemented consistently across all study participants?	✓	X
Index test		
Were the u‐EPX results interpreted without knowledge of the results of the reference standard?	✓	X
Outcome measures		
Was there a clear cut criteria specified for the diagnosis of asthma?	✓	X

Note: The checkmark represents the desired quality of the study for each quality assessment question.

### Statistical analyses

The meta‐analysis was performed using Stata software. The effect size representing the difference in u‐EPX levels between asthmatics and control group children were calculated using standardized mean differences (SMD), also referred to as *Cohen's d*. For studies that did not report the measures as means and standard deviations, attempts to contact the authors for the actual data were unsuccessful. For uniform measures of u‐EPX to compare, evidence based conversion formulas 14, 15, 16 were employed to calculate estimated means and standard deviations from the reported values (e.g., median values, quartiles, and ranges).

## Results

### Study characteristics

As of August 2015, there were 27 studies which investigated the relationship between EPX and childhood asthma. Six studies used serum measures of EPX 11, 17, 18, 19, 20, 21, 18 studies used urinary measures of EPX 22, 23, 24, 25, 26, 27, 28, 29, 30, 31, 32, 33, 34, 35, 36, 37, 38, 39, and three studies reported both serum and urinary measures of EPX 10, 12, 40 in relation to asthmatic and non‐asthmatic status. There were 13 longitudinal studies 10, 17, 18, 22, 23, 24, 26, 27, 28, 32, 33, 34, 39, nine cross‐sectional studies 11, 12, 25, 30, 31, 35, 36, 37, 38, and two case–control studies 19, 40 (Table 2). In three of the studies, among the participants recruited for cross‐sectional data, some were followed longitudinally for a time period 20, 21, 29.

**Table 2:** Studies investigating eosinophil protein X (u‐EPX, serum EPX) and childhood asthma

Author	Objective/hypothesis	Study design	Study sample	Comparison group	Diagnosis of asthma	Results (u‐EPX µg/mmol Cr; serum EPX µg/L)	Conclusions	Disadvantages
Carlstedt (2011), Sweden 22	To determine if u‐EPX and exhaled nitric oxide (FeNO) are objective markers of early airway inflammation in infants	Prospective cohort	110 mother–infant pairs with infant age 2–6 months		Based on parents completed questionnaire and physicians’ examination	No difference in u‐EPX/c levels in infants with a history of wheezing or atopic heredityu‐EPX levels were higher in mothers with self‐reported asthma (*P* = 0.09).	The use of u‐EPX as a marker of early inflammation was not supported with this sampling method	Sampling method for urineSamples were collected by parents using cellulose tissue sanitary towels put into diapers
Chawes (2011), Denmark 23	Elevated levels of u‐EPX, uLT, and u11β‐PGF_2α_ early in life reflects pre‐symptom disease activity preceding later development of atopic disease	Prospective	369 healthy at risk neonates (those born to mothers with asthma)		Based on physicians’ objective assessment and analysis of symptom recordings completed by parents	u‐EPX was not associated with an increased risk of developing asthma by age 7 years, odds ratio 1 (95%CI 0.5–1.8, *P* = 0.99).	u‐EPX measured in healthy asymptomatic 1‐month‐old neonates was associated with development of allergic sensitization, nasal eosinophilia, and eczema during pre‐school age but not asthma	For generalization of results, high risk nature of cohort is a limitation
Gore (2003), United Kingdom 24	To investigate the relationship between u‐EPX and clinical phenotypes suggestive of allergic diseases	Prospective	903 children at age 3 years, followed prospectively from birth		Based on physicians’ objective assessment and analysis of symptom recordings completed by parents	Physician diagnosed asthma was strongly associated with u‐EPX levels, with highest levels found in children receiving asthma medication (*P* < 0.001)Children receiving short‐acting β‐agonists had significantly higher U‐EPX levels (median 93.1, 95%CI, 79.5–109.0) compared with those who did not (70.5, 95%CI, 67.0–74.3 (*P* = 0.001).	u‐EPX level reflects the presence of atopy and associated symptomsIts role as an adjunct in the diagnosis and management of allergic airway disease, in particular cough‐variant asthma in early childhood might be important	Circadian variation in the sample selection
Gravesande (1999), Germany 25	To evaluate the usefulness of EPX excretion in monitoring therapy in asthmatic children	Cross‐sectional	22 stable asthmatic children 21 chronic asthmatics 7 acute asthmaticsAge 3.6–16.1 years	16 non‐atopic, non‐asthmatic controls of ages 4.4–18.8 years	Diagnosis of asthma (chronic vs. acute) was based upon a history of chest tightness, cough and dyspnea and a 12% improvement of FEV_1_ and/or FVC after β_2_ agonists	Significantly higher u‐EPX in chronic asthmatics (mean 124.7 ± SD 84.6; *P* *= *0.0009) and in acute asthmatics (233.3 ± 174.5; *P* *= *0.0006) before treatment compared with non‐atopic, non‐asthmatic controls (53.4 ± 29.0).u‐EPX decreased significantly in chronic asthmatics after 6 weeks of treatment with inhales corticosteroids (*P* = 0.02), and in acute asthmatics 3 months after discharge (*P* = 0.02).	u‐EPX is a valid tool for monitoring the effects of anti‐inflammatory therapy in asthmatic children	
Hoekstra (1996), Netherland 40	To whether serum and urine concentrations of eosinophil‐derived proteins in children could be related to the diagnosis of asthma	Case–control	22 children of age 4–14 years, having diagnosis of allergic asthma with perennial symptoms	17 healthy controls having no symptoms or signs of asthma or allergy	Based on American Thoracic Society criteria	Higher serum EPX levels among asthmatics (median 9.45; IQR 6.2–12.7) when compared to healthy controls (median 2.9; IQR 1.2–5.9).Higher urinary EPX levels among asthmatics (median 162; IQR 91–200) when compared to healthy controls (median 55; IQR 34–79).	u‐EPX is a more complete reflection of the eosinophil cell.u‐EPX could be a simple, noninvasive, and less invasive alternative in the diagnosis of childhood asthma	Effect of confounders like asthmatic medications has not been accounted for
Kalaajieh (2002), Lebanon 26	To determine the concentration u‐EPX to predict the severity and activity of asthma in children	Longitudinal	80 non‐atopic asthmatic children aged 5–12 years	25 healthy, age and sex matched controls	Based on medical history and complete physical examination at hospitalSeverity of asthma based on asthma score including clinical and physiological parameters	u‐EPX levels were significantly higher in asthmatics, both during (139.6 ± 11.7) and after (66.5 ± 9.3) attacks.u‐EPX concentrations were significantly higher in asthmatic children than controls (*P* < 0.001)The change in u‐EPX levels among mild, moderate, and severe asthmatics during attack and 2 weeks after attack were statistically significant with *P* < 0.05.u‐EPX was highest in patients with severe asthma during attack (191.5 ± 11.3) and at the 2 week follow‐up (103.8 ± 9.4).	Statistically significant concentrations of u‐EPX in asthmatic children, especially during acute exacerbationMeasurement of u‐EPX concentration may be useful in quantifying bronchial inflammation, thus, may serve as a marker of severity of the disease exacerbation and also facilitate early diagnosis and staging of the disease	
Kim (2007), Korea 17	To evaluate the use of serum EPX as a marker of airway inflammation in asthma in the diagnosis and evaluation of the severity and bronchial hyper‐responsiveness in childhood asthma	Longitudinal	72 atopic asthma children aged 6–15 years36 children with non‐atopic asthma	43 age‐matched healthy children	Current asthma: wheezing or cough (absence of cold) in the past 12 months and a physician's diagnosis (any time in the patient's history)The severity of asthma was defined based on episodes of wheezing per year, speech interruption, and nocturnal wakening due to wheezing	Serum EPX levels were higher among atopic asthmatics (80.1 ± 34) compared to non‐atopic asthmatics (60.4 ± 36.3), and controls (52.8 ± 34.4) (*P* < 0.001).Serum EPX did not differ among non‐atopic asthmatics and controls.Serum EPX levels of children with severe asthma (96.8 ± 31.7) differed significantly from those of children with mild (61.6 ± 35.6, *P* < 0.001) or moderate (69.7 ± 32.6, *P* < 0.01) asthmaThere was no statistical difference between the EPX levels of the mild and moderate asthma groups	Serum EPX can be clinically useful in atopic asthma childrenSerum EPX might be another supportive biomarker for the diagnosis of atopic asthma, evaluation of asthma severity, and assessment of bronchial hyper‐responsiveness	
Kim (2010), Korea 18	To evaluate the utility of the serum EPX as a marker of eosinophil degranulation and its possible correlation with disease severity in childhood asthma	Longitudinal	43 children with asthma aged 1.4–5 years having acute asthmatic symptomsAll were atopic with at least one positive skin prick test	19 age matched normal controls without history of acute or chronic respiratory symptoms	Diagnosed based on the Global Initiative for Asthma Guidelines	Serum EPX levels were significantly higher among asthma group when compared to controls (median 20, IQR 13.8–38.3), during both the acute (80, 55.2–113.0) (*P* < 0.0001) and stable (42.9, 28.8–79.2 (*P* < 0.0001) phases, and were also significantly elevated when comparing the acute phase to the stable phase (*P* < .001).Within the asthma group, the serum EPX levels significantly correlated with clinical asthma scores (*r* = 0.850, *P* < 0.0001)Serum EPX levels were significantly higher in the severe subgroup when compared to moderate (*P* < 0.0001) and mild subgroups (*P* < 0.0001)	Serum EPX is a useful marker for identifying disease activity in asthmatic childrenSerum EPX levels may better reflect disease severity than ECP levels or total eosinophil count	Selection of subjects is based on the need for hospitalization and thus the sample is not representative of all asthma patients
Koller (1999), Austria 12	To investigate whether eosinophil granule proteins correlated and whether there is a relationship between disease activity, pulmonary function and bronchial hyper‐reactivity	Cross‐sectional	28 children with mild to moderate, atopic bronchial asthma (mean age 11.1 ± 2.2 years) under current anti‐asthmatic treatment	11 healthy non‐atopic and non‐smoking smoking adults (mean age 23.5 ± 2.24 years)	Based on recurrent obstructive pulmonary symptoms that were reversible with β_2_ agonists and after exclusion of other conditions	u‐EPX levels significantly raised in asthmatic children (median 49.4; IQR 34.2–64.0) compared to healthy controls (16.5, 7.4–25.6) (*P* = 0.0001)u‐EPX were significantly higher in symptomatic patients than in asymptomatic patients (*P* < 0.03)	Eosinophils activation in mild to moderate asthma as reflected by serum and urine concentrations of EPX and ECP is related to disease activity and weakly, albeit significantly to pulmonary function.Supports the use of anti‐inflammatory markers (serum EPX, serum ECP and u‐EPX) in monitoring the asthmatic children than pulmonary function	Discontinuation of topical steroids 48 hours prior to the tests may not be sufficient to wear off the anti‐inflammatory effect
Kristjansson (1996), Sweden 10	To investigate increased amounts of eosinophil granulocyte proteins in urine and serum reflect ongoing asthmatic inflammation and whether decreasing values reflect successful treatment	Longitudinal	12 children with atopic asthma aged 8.1–15.6 years	9 children without any atopic or known other diseases aged 9.1 to 15.4 years	Grading of asthma severity based on frequency of symptoms and the treatment requiredAtopy was confirmed with a positive skin prick test and/or positive serum RAST and increased IgE	At baseline, u‐EPX was significantly higher in children with atopic asthma (mean 116.4) than in the control subjects (mean, 43.0) (*P* = 0.004)In the asthma group, u‐EPX decreased significantly after 3 months of treatment with budesonide (116.4–68.4) (*P* = 0.005)	u‐EPX and serum ECP levels are useful markers of eosinophil activation and ongoing inflammatory activity in children atopic asthmaThese levels are significantly lowered with anti‐inflammatory treatment with inhaled steroids	
Labbe (2001), France 27	To analyze the role of u‐EPX as a biomarker of eosinophil activation in asthmatic children	Longitudinal	88 asthmatic children of age 1.1–16.1 years	34 children without any respiratory or atopic disorders	Diagnosed by pediatric pulmonologist	At baseline, u‐EPX is significantly higher in asthmatics children (171) than in control group (60) (*P* < 0.01)Among asthmatics, there was no difference in u‐EPX levels among atopic and non‐atopic childrenAfter 3 months of corticosteroid therapy, there was a significant decrease in u‐EPX levels in asthmatics of recent onset who were previously not on steroid treatment (−54, *P* < 0.02).	Measurement of u‐EPX, a reliable, non‐invasive, sensitive and reproducible method to assess bronchial inflammationu‐EPX levels useful in‐management of chronic bronchial disease among infants‐ studies of effectiveness of anti‐inflammatory treatment‐ Assessment of compliance with pharmacologic therapy	
Lonnkvist (2001), Sweden 28	To relate clinical symptoms and deterioration of childhood asthma to inflammatory markers, after withdrawal of inhaled corticosteroids	Longitudinal	34 mild‐to‐moderate asthmatic children aged 9–16 years on budesonide treatment selected to continue or discontinue treatment	16 age matched healthy controls		Baseline values of serum EPX and urine EPX were significantly lower in healthy controls compared to asthmatics (*P* < 0.001)Lower baseline levels of u‐EPX (126) were associated with lower risk of exacerbations after withdrawal of budesonide (RR = 0.44, *P* < 0.03)Among symptomatic asthmatics, those with treatment withdrawal had higher u‐EPX levels (median 191, range 50–426) compared to those with continued treatment (median 135, range 68–435)	Because of easy sampling, EPX can be preferred marker in distinguishing healthy children from those with airway inflammation when diagnosis is unclear due to vague and atypical symptoms	
Lugosi (1997), Austria 29	To determine clinical use of u‐EPX in monitoring airway inflammation in childhood asthma	Cross‐sectional followed by longitudinal follow‐up for some of the participants	80 children with bronchial asthma of age 10.1 ± 3.1 years	24 healthy, age‐matched controls.Age = 11 ± 3.9 years	Based on obstructive pulmonary symptoms which are reversible with β_2_ agonists	u‐EPX levels were increased in asthmatic children (median 68.4) compared to healthy controls (median 35.3) Cr; *P* < 0.0001).u‐EPX levels were higher in symptomatic (median 123.5) than in asymptomatic patients (median 48.9) (*P* < 0.0001) independent of treatment or atopy.	Measurement of u‐EPX can be an alternative to assess asthma activity in children aged less than 5 years	
Mattes (1999), Germany 30	To assess the relationship between u‐EPX and other markers of airway inflammation in corticosteroid‐dependent childhood asthma	Cross‐sectional	25 children with stable asthma of ages 6–16 years on corticosteroidsids	9 healthy controls without atopic disorders or sensitization to allergens	Based on clinical symptoms (cough, wheeze, and/or dyspnea) and increase in FEV1 with bronchodilator	u‐EPX levels significantly higher in asthmatics (median 58.2, 90%CI 29.2–181.1) compared to healthy controls (median 30.6, 90%CI 20.8–75.5) (*P* = 0.02)	u‐EPX levels are significantly correlated with exhaled NO levels in asthmatics	
								
Nuijsink (2007), Netherlands 31	To investigate the relationship between u‐EPX and asthma symptoms	Cross‐sectional	180 atopic children of ages 6–16 years with moderately severe asthma		From medical records	u‐EPX levels were measured as median 185 and range 2–3114.	u‐EPX levels did not correlate with established markers of asthma severity and eosinophilic airway inflammation in atopic asthmatic children	Diurnal variability may have introduced scatter of u‐EPX, thus weakening a possible correlation
Nuijsink (2013), Netherlands 32	Changes in u‐EPX would be related to changes in eosinophilic airway inflammation	Longitudinal	205 atopic asthmatic children of ages 6.4–16.8 years using inhaled fluticasone		From medical records	After 2 year treatment period, the geometric mean u‐EPX significantly decreased from 159 to 104 (*P* < 0.001)No correlations were found between u‐EPX and asthma symptoms.u‐EPX levels were higher in patients with airway hyper‐responsiveness (AHR) than in patients without AHR	u‐EPX seems unlikely to be useful biomarker for monitoring asthma in an individual child	
Oymar (2001), Norway 35	To determine the clinical value of measuring u‐EPX in children with asthma and to evaluate the influence of atopy and airway infections	Cross‐sectional	170 children with asthma of ages 12–179 months	64 healthy controls	Based on episodes of cough/wheeze (response to β_2_ agonists) persisting or recurring for at least 6 months	Compared to healthy controls (median 54, IQR 40–89), u‐EPX levels were elevated in children with acute asthma (median 132, IQR 77–195, *P* < 0.001) and chronic asthma (median 93, *P* < 0.01)Among acute and chronic asthmatics, atopic children had higher levels of u‐EPX than non‐atopics (*P* < 0.05)Among chronic asthmatics, u‐EPX levels were similar in asymptomatic and symptomatic asthmatics	u‐EPX may reflect differences in eosinophil involvement between children atopic and non‐atopic asthma. However, the individual spread within groups and the influence of airway infect limits its clinical use in childhood asthmau‐EPX alone is not sufficient to describe the airway inflammation or symptom activity in the individual child	Circadian variation in u‐EPX has not been accounted for as the samples were distributed throughout 24‐h period
Oymar (2001), Norway 34	To determine the role of u‐EPX in the prediction of recurrent wheezing and allergic sensitization 20 months later	Longitudinal	105 children of ages 1–12 months hospitalized for wheezing			u‐EPX was not a predictive factor for recurrent wheezing (OR = 1, 95%CI = 0.99–1.01)	Study demonstrated that u‐EPX cannot predict recurrent wheezing 20 months after the first hospitalization and therefore might have limited role in the prediction of asthma	Both under‐ and over‐reporting of symptoms because of recall bias and differences in threshold for symptom self‐reporting
Oymar (2001), Norway 33	To evaluate the ability of u‐EPX and eosinophil counts to predict persistent and atopic asthma, 2 years after hospitalization for acute asthma	Longitudinal	32 children of ages 12–36 months who were hospitalized for acute asthma	20 healthy children of ages 10–51 months	Diagnosed by pediatrician	On admission, u‐EPX levels were higher in asthmatic children (median 120 μg/mmol Cr, IQR 67–123) than in controls (median 60, IQR 38–74) (*P* < 0.001)At follow‐up, u‐EPX levels were higher in those with persistent atopic asthma (median 173, IQR 123–196), than those with persistent non‐atopic asthma (median 73, IQR 46–105) (*P* < 0.05), and those with transient asthma (median 106, IQR 42–167) (*P* < 0.05)u‐EPX was the only parameter able to predict persistent atopic asthma (*P* = 0.03)	Results suggest a possible role for u‐EPX in the prediction of persistent atopic asthma when measured during active symptoms in young asthmatic childrenIt might be possible for u‐EPX to be employed in combination with other parameters to predict the outcome of asthma in early childhood	Study included only a small number of children, and larger studies with a longer follow‐up are needed to confirm the results
Rao (1996), United Kingdom 11	To assess the role of serum EPX and ECP levels as measures of airway inflammation in childhood asthma	Cross‐sectional	48 children of ages 5–10 years with moderately severe asthma		Diagnosed by physicians	serum EPX negatively correlated with FEV_1_ (*r* = −0.35, *P* = 0.01) and FEF_25–75%_ (*r* = −0.36, *P* = 0.01)No data pertaining to the levels of serum EPX are available in the study	Serum markers of eosinophils correlate with airway function in childhood asthma Similar to adult asthma	
Reichenberg (2000) Sweden 36	To examine this relationship between asthma severity u‐EPX in children	Cross‐sectional	61 children of ages 7–9 years with asthma	Healthy children from Lugosi study		Median u‐EPX among asthmatic children was 88.6 (95%CI 67.5–135.7)In healthy children of the same age group, Lugosi et al. found median u‐EPX levels of 35.3 (95%CI 25.9–50.2) with the same method of analysis	Findings give no further support for applying u‐EPX as a general measure of disease severity in childhood asthma	Study and control groups are from different populations to be compared
Remes (1998), Finland 19	To determine the value of measuring serum EPX and ECP in diagnosing childhood asthma	Case–control	36 asthmatic children of ages 7–12 years	166 children without asthma	Diagnosed by pediatric allergist clinically and by objective tests	Serum EPX levels were higher in asthmatics not receiving anti‐inflammatory therapy (median 59.9, IQR 33.6–99.2) compared to controls (median 26.2, IQR 19.2–40.1) (*P* < 0.001)Elevated serum EPX was significantly associated with asthma (OR = 2.61, 95%CI 1.19–5.74	The presence of asthma raises serum levels of EPX and ECP in childrenConcomitant existence of atopic sensitization and allergic diseases also raises serum ECP and EPXSerum EPX and ECP can only be useful in relation to whole clinical situation in childhood asthma	This study does not assess severity of asthma
Severien (2000), Germany 37	To compare levels of urinary EPX and leukotriene E4 between children with stable atopic asthma (different disease severity) and healthy controls	Cross‐sectional	80 children of ages 10.5 yrs ± 2.5 years with asthma	28 healthy controls matched for age and sex	Diagnosed by physician	u‐EPX was significantly increased in asthmatic children (median 85.5, IQR 64–131.5, SD 76.2) compared with controls (median 48.5, IQR 43.2–90, SD 112.1) (p = 0.006)No differences in u‐EPX between the group of mild and the group of moderate to severe asthmatic children	Urinary EPX is a useful noninvasive marker of airway inflammation and can be helpful as a complementary test in guiding asthma management	
Tauber (2000), Austria 38	To evaluate the use of u‐EPX in epidemiologic studies in identifying atopic and asthmatic children	Cross‐sectional	877 Austrian school children if age 10–12 years		Based on modified International Study on Asthma and Allergy in Childhood Questionnaire completed by parents	u‐EPX levels were higher in children with physician‐diagnosed asthma (median 142.8 µg/mmol Cr) compared to healthy controls (median 63.9) (*P* < 0.0001).The odds ratio for u‐EPX levels over the 90th percentile was significantly elevated for asthma (6.10, 95%CI 2.44–15.24)	Great overlap between controls and symptomatic asthmatics reduces the sensitivity of u‐EPX in determination of the prevalence of asthma in epidemiologic studies	
Wojnarowski (1999), Austria 39	To study the relationship between levels inflammatory markers (EPX and ECP in urine and nasal fluid) and clinical severity of childhood asthma	Longitudinal	14 children of age 7.01–15.08 years with mild persisting asthma		Based on National Heart, Lung, and Blood Institute Criteria	Mean u‐EPX levels at the last visit before exacerbation was 46.4 (SD 28), and at first visit after exacerbation was 46.1 (SD 23.5)Mean EPX values before, at, and after exacerbation were not different from values of patients without exacerbation at any time pointFor children treated with long acting B2 agonists there was no difference in u‐EPX compared with children without this therapy	Though an increase in inflammatory markers during an exacerbation is seen, there exists a great variability in ECP and EPX levels in each patient and no increase in u‐EPX (any inflammatory markers) preceding an exacerbation	Very small sample size to draw conclusions
Zimmerman (1993), Sweden 21	To examine serum EPX, ECP, and eosinophil counts to distinguish between symptomatic and asymptomatic asthma patients, independent of treatment	Cross‐sectional followed by longitudinal follow‐up for some of the participants	34 asthmatic children of age 6–17 years	13 age matched children with chronic urticarial but no asthma	Symptomatic asthma: cough, wheeze, or breathlessness, physical symptoms of rhonchi and decreased air entry on auscultation and at least 15% response to inhaled bronchodilator	At baseline, serum EPX levels were higher in symptomatic asthmatics (mean 54.4) compared to control group (mean 23.5).Serum EPX levels significantly decreased among symptomatic asthmatics following treatment (54.4 to 24.5, *P* < 0.001), while the change among asymptomatic children were not significant	Serum EPX levels were higher in symptomatic asthmatics compared to asymptomatic asthmatics	
Zimmerman (1994), Sweden 20	To determine relationship between serum EPX and ECP, and atopy‐related symptoms in asthmatic children less than 5 years, before and after inhaled steroid use	Cross‐sectional followed by longitudinal follow‐up for some of the participants	14 atopic asthmatic children of age <5 years	13 non‐atopic asthmatic children of age <5 years		At baseline, serum EPX were higher in atopic asthmatics (mean 69.0) than non‐atopic asthmatics (mean 19.6) (*P* < 0.01)After treatment with inhaled steroids, the difference in serum EPX levels between symptomatic atopic asthmatics (mean 89.3) and non‐atopics (mean 26.7) was not statistically significant	Higher levels of serum EPX among atopic asthmatics compared to non‐atopic asthmatics were observed only during symptomatic phase. Upon treatment this difference became non‐significant	

### Sample size and age

The sample sizes and ages of children for all the studies are displayed in Table 3. Children's ages varied greatly from birth to 18 years, and the sample sizes ranged from small (*n* = 14) to large sizes (*n* = 903). Sources of patients consisted of patients in the emergency room 33, 34, 35, children from outpatient departments 11, 12, 17, 20, 23, 25, 26, 27, 29, 30, 35, 38, 39, 40, children recruited from school 38, a mother–infant cohort 22, hospitalized inpatients 18, and birth cohorts 22, 23.

**Table 3:** Study methodologies for measuring u‐EPX

Author	Sample size	Age group	Test kit	Detection limit	Spin down sample?	Freezer temperature (−°C)	Duration before freezing	Other info
Carlstedt (2011) 22	110	2–6 months	ELISA immunoassay (Diagnostics Development, Uppsala, Sweden)				Tubes were kept cold and frozen within 24 h	Urine collected by parents from sanitary towels made of cellulose tissue placed in diaper
Chawes (2011) 23	369	0–7 years	Double‐antibody immunoassay (RIA − Pharmacia, Uppsala, Sweden)	<3 μg/L		80	Immediately	Transferred to 3.6 mL Nunc tubes and aliquots stored without addition of any preservatives
Gore (2003) 24	903	3 years	Specific RIA (Pharmacia Diagnostics AB)	<3 μg/L		20	Within 10 h	Diluted ×11 in phosphate buffer
Gravesande (1999) 25	44	3–18 years	RIA (Pharmacia & Upjohn, Freiburg, Germany)	<3 μg/L		70	Immediately	
Hoekstra (1996) 40	39	4–14 years	RIA (Pharmacia, Uppsala, Sweden)		Serum centrifuged twice at 1450*g*	20		
Kalaajieh (2002) 26	105	5–14 years	Specific RIA (Pharmacia, Uppsala, Sweden)	<3 μg/L		20	Within 10 h	
Kim (2007) 17	151	6–15 years	ELISA kit (7630, MBL, Nagoya, Japan)					Serum on micro well with anti‐human EPX antibody
Kim (2010) 18	62	1.4–5 years	ELISA (MBL, Woburn, MA)	<0.62 ng/ml	Serum centrifuged twice at 1350*g*	70	1 h at 25°C	
Koller (1999) 12	28	11.1 ± 2.2 years	Sensitive RIA (Pharmacia Upjohn AB, Uppsala, Sweden)			70	Immediately	Diluted 11× in phosphate buffer
Kristjansson (1996) 10	21	8.1–15.6 years	Specific competitive RIA (Pharmacia, Uppsala, Sweden)	<3 μg/L		20	Immediately	
Labbe (2001) 27	100	1.1–16.5 years	RIA (Pharmacia Diagnostics, Uppsala, Sweden)	<3 μg/L		20		Diluted in phosphate buffer
Lonnkvist (2001) 28	50	9–16 years	Specific RIA (Pharmacia & Upjohn)					
Lugosi (1997) 29	104	10.1 ± 3.1 years	Specific RIA (Pharmacia, Uppsala, Sweden)	<3 μg/L		20	Immediately	Diluted 11× in phosphate buffer
Mattes (1999) 30	34	6–16 years	Double antibody RIA (Pharmacia & Upjohn)			70	Immediately	
Nuijsink (2007) 31	180	6–16 years	ELISA (MBL, Nakaku Nagoya, Japan)	<0.62 μg/L		20		50‐fold diluted sample
Nuijsink (2013) 32	288	6.4–16.8 years	ELISA (MBL, Nakaku Nagoya,Japan)	<0.62 μg/L		20	Immediately	50‐fold diluted sample
Oymar (2001) 33	52	10–51 months	Pharmacia Diagnostics Uppsala, Sweden	<3 μg/L		20	Within 10 h	
Oymar (2001) 34	105	1–12 months	Specific competitive RIA (Pharmacia, Uppsala, Sweden)	<3 μg/L		20	Acute asthmatics: within 10 hChronic asthmatics: within 6 h	
Oymar (2001) 35	314	0–15 years	RIA (Pharmacia & Upjohn Ab, Uppsala, Sweden)	<3 μg/L		20	Within 10 h	
Rao (1996) 11	48	5–10 years	RIA (Pharmacia & Upjohn Ab, Uppsala, Sweden)	<3 μg/L	Centrifuged at 3500 RPM for 10 min	70		
Reichenberg (2000) 36	85	7–9 years	RIA (Pharmacia)					
Remes (1998) 19	235	7–12 years	Pharmacia Diagnostics Uppsala, Sweden	>38.5 μg/L for the upper limits of normality	Serum centrifuged 1000–1350*g* for 10 min, serum separated and aliquoted	70	Serum allowed to clot 60–120 min	
Severien (2000) 37	108	10.5 ± 2.5 years	Double antibody RIA (Pharmacia Sweden)	<3 μg/L	Centrifuged to remove cellular debris at 10,000*g* for 8 min	70		
Tauber (2000) 38	877	10–12 years	RIA (Pharmacia & Upjohn Ab, Uppsala, Sweden)			30	Within 3 h	Samples diluted ×11 in phosphate buffer
Wojnarowski (1999) 39	14	7.1–15.8 years	Specific competitive RIA (Pharmacia, Uppsala, Sweden)	<3 μg/L	Centrifuged twice for 10 min at 1200*g*	70	Immediately	Samples diluted ×11 in phosphate buffer
Zimmerman (1994) 20	27	0–5 years	RIA (Kabi Pharmacia Diagnostics AB)					
Zimmerman (1993) 21	30	5.6–18 years	Pharmacia Diagnostics AB, Uppsala, Sweden					

### Diagnosis and treatment of asthma

More than one‐half of the studies conducted at least one type of pulmonary test on young participants, including whole‐body plethysmography 12, 24, 29, spirometry 10, 11, 17, 21, 28, 30, 31, 32, 35, 37, 40 PEFRs 21, 26, 36, 37, 39, bronchial hypersensitivity using methacholine challenge 12, 17, 28, 31, 32, 40, FE(NO) levels in a subsample 22, 30, 31, 32, FEV_1_ 11, 17, 21, 27, 28, 29, 30, 31, 35, 37, 39, 40, and FEF 25–75% 11, 27.

Skin prick tests (SPT) were used in four studies 10, 12, 17, 18, 20, 24, 35 to test for atopy. Questionnaires on children's wheezing were completed by parents 2, 36 or young patients 38. In addition, seven studies used respiratory diary cards 11, 20, 28, 31, 32, 39, 40. Only one study focused on quality of life, using the Pediatric Asthma Quality of Life questionnaire and International Study on Asthma and Allergy in Childhood questionnaire 36.

### Measurement of EPX

Differences in urinary EPX levels could be influenced by the methodology employed for measuring urinary EPX. Table 3 summarizes the test kit, detection limits, centrifuge spinning information (for serum not urine), freezer temperature, and duration of time before freezing and other information. Twenty‐two studies used Pharmacia's radioimmunoassay 10, 11, 12, 19, 20, 21, 23, 24, 25, 26, 27, 28, 29, 30, 33, 34, 35, 36, 37, 38, 39, 40, and five studies used ELISA immunoassay (from Diagnostic Development, Uppsala, Sweden and Medical and Biological laboratories, Nagoya, Japan) 17, 18, 22, 31, 32. Eleven studies froze their samples at −20°C 10, 24, 26, 27, 29, 31, 32, 33, 34, 35, 40, eight studies froze samples at −70°C 11, 12, 18, 19, 25, 30, 37, 39. The duration before freezing was the only factor that differed considerably, and could account for some of the variation. Carlstedt et al. 22 kept their samples cold and froze them within 24 h. Gore et al., Kalaajieh et al., Oymar, and Oymar et al. froze their samples within 10 h 24, 26, 33, 35, while Tauber et al. froze samples within 3 h 38. Chawes et al., Gravesande et al., Koller et al., Kristjansson et al., Lugosi et al., Mattes et al., Nuijsink et al., and Wojnarowski et al. froze their samples immediately 10, 12, 23, 25, 29, 30, 32, 39.

### Qualitative analysis: preliminary cut points of u‐EPX

Urine eosinophil protein X concentrations were correlated with a variety of outcomes (endpoints) (Table 4). For example, u‐EPX concentrations were higher in all asthmatic children compared to controls 18, 21, 25, 26, 27, 29, 30, 33, 35, 36, 37, 40 and higher during attacks in atopic 10, 19, 20, 29, 32, 33, 35, 38 and non‐atopic asthmatics 19, 20, 29, 33, 35, 38 compared to controls. Table 4 summarizes the reported values of EPX from all the included studies.

**Table 4:** EPX concentration ranges

Studies reporting only urinary EPX levels							
	Asthmatics	Asymptomatic	Atopic	Non‐atopic	Acute	Chronic	Healthy controls
Carlstedt (2011) 22	NA	NA	NA	NA	NA	NA	Median: 21.4Range: 7.1–81.1IQR: 15.5
Chawes (2011) 23	NA	NA	NA	NA	NA	NA	NA
Gore^‡^ (2003) 24	87.78 (77.38–99.58)	NA	NA	NA	NA	NA	NA
Gravesande^+^ (1999) 25	NA	NA	NA	NA	233.3 ± 174.5	124.7 ± 84.6	53.4 ± 29.0
Kalaajieh^+^ (2002) 26	NA	66.5 ± 9.3	NA	NA	139.6 ± 11.7Mild attack:88.2 ± 7.2Moderate attack: 119.6 ± 8.5Severe attack: 191.5 ± 11.3	NA	35.3 ± 6.2
Labbe* (2001) 27	Mild/moderate: 171 (146–196)	NA	NA	NA	NA	NA	60(44–76)
Lonnkvist* (2001) 28		NA	NA	NA	NA	NA	68 (31–204)
Lugosi* (1997) 29	68.4 (41.5–115.0)	48.9 (30.8–67.7)	65.1 (37.7–118.0) Symptomatic: 131.5 (86.0–208.5)	86.0 (48.7–112.1) Symptomatic: 108.8 (88.2–145.5)	123.5 (86.5–192.8)	NA	35.3 (25.9–50.2)
Mattes§ (1999) 30	NA	58.2 (29.2–181.1)	NA	NA	NA	NA	30.6 (20.8–75.5)
Nuijsink^¶^ (2007) 31	185.0 (2.0–3114.0)	NA	NA	NA	NA	NA	NA
Nuijsink^¶^ (2013) 32	NA	NA	184 (2–3114)	NA	NA	NA	NA
Oymar* (2001) 33	120 (67–123)	NA	173 (123–196)	73 (46–105)	NA	NA	60 (38–74)
Oymar* (2001) 34	NA	NA	NA	NA	NA	NA	NA
Oymar* (2001) 35	NA	NA	Acute 155 (113–253)Chronic 110 (65–162)	Acute 102 (56–168)Chronic 60 (39–123)	132 (IQR 77–195)	93 (IQR 46–149)	54 (IQR 40–89)
Reichenberg* (2000) 36	88.6 (67.5–135.7)	NA	NA	NA	NA	NA	Used results from Lugosi 35.3 (25.9–50.2)
Severien* (2000) 37	85.5 (64–131.5)SD 76.2Mild/moderate: 84 (54.5–131.5)Severe: 91 (65–158)	NA	NA	NA	NA	NA	48.5 (43.2–90) SD 112.1
Tauber^§^ (2000) 38	NA	NA	89.6 (27.6–280.3)	62.5 (22.2–174.8)	NA	NA	63.6 (28.76–140.96)
Wojnarowski (1999) 39	NA	NA	NA	NA	NA	NA	NA
Studies reporting only serum EPX levels							
Kim^+^ (2007) 17	NA	NA	NA	NA	Mild attack: 76.92 ± 36.2Moderate attack: 98.07 ± 27.87Severe attack: 107.3 ± 10.76	NA	NA
Kim* (2010) 18	NA	NA	NA	NA	80 (55.2–113.0)	42.9 (28.8–79.2)	20 (13.8–38.3)
Rao (1996) 11	NA	NA	NA	NA	NA	NA	NA
Remes* (1998) 19	NA	NA	47.4 (34.8–97.2)	Mean: 86.6	NA	NA	26.2 (19.2–40.1)
Zimmerman (1994) 20	NA	NA	Mean:69Asymptomatic: 42Symptomatic: 89.3	Mean: 19.6Asymptomatic: 23.9Symptomatic: 23.9	NA	NA	NA
Zimmerman (1993) 21	NA	NA	NA	NA	Mean 54.4	Mean 35.3	Mean 31.2
Studies reporting both urinary and serum EPX levels							
Hoekstra* (1996) 40	NA	u‐EPX 162 (91–200) serum EPX 31.8 (23.3–40.7)	NA	NA	NA	NA	u‐EPX 55 (IQR 34–79)Serum EPX 15.4 (IQR 10.3–23)
Koller* (1999) 12	NA	NA	Serum EPX 74.8 (40.2–101.2)u‐EPX 49.4 (34.2–64.0)	NA	NA	NA	Serum EPX 24.3 (22.3–29.2)u‐EPX 16.5 (IQR 7.4–25.6)
Kristjansson^‡^ (1996) 10	NA	NA	Serum EPX 94.7 (68.2–121.3)u‐EPX 116.4 (71.2–161.6)	NA	NA	NA	Serum EPX 30.8 (22.1–39.5) u‐EPX 43 (23.3–62.7)

Note: *Represents values reported as “median (quartiles 1 and 3)”; ^+^represents values as “mean ± standard deviation”; ^‡^represents values as “mean (95% confidence interval)”; ^§^represents values as “median (90% confidence interval)”; ^¶^represents values as “median (range).”

Studies reported levels of u‐EPX in a variety of groups of asthmatic children (e.g., chronic asthmatics, chronic asymptomatic asthmatics, chronic symptomatic asthmatics, atopic asthma, atopic symptomatic asthma, atopic asymptomatic asthma etc.) which limited the comparison of u‐EPX levels across the studies. Mean urinary EPX levels were comparable between the acute asthmatics groups of Kalaajieh (Mean 139.6 ± SD 11.7) 26, Lugosi (Estimated mean 134.27 ± SD7 9.6) 29, and Oymar (estimated mean 134.67 ± SD 87.4) 35.

Healthy comparison groups were identical for Hoeskstra (estimated mean 56 ± SD 34.05 μg/mmol Cr) 40, Labbe (Mean 60 ± SD 8 μg/mmol Cr) 27, Oymar (estimated mean 57.3 ± SD 27.13 μg/mmol Cr) 33, Oymar (estimated mean 61 ± SD 36.65 μg/mmol Cr) 35, and Severien (estimated mean 60.57 ± SD 36 μg/mmol Cr) 37. Additionally, a group of three studies reported identical mean u‐EPX levels among the healthy comparison groups including Kalaajieh (mean 35.3 ± SD 6.2 μg/mmol Cr) 26, Lugosi (estimated mean 37.133 ± SD 18.28 μg/mmol Cr) 29, and Kristjansson (mean 43 μg/mmol Cr) 10. Mean u‐EPX levels among the healthy controls reported by Lonnkvist (estimated mean 75.75 ± 43.25 μg/mmol Cr) 28, and Koller (estimated mean 16.5 ± SD 13.92 μg/mmol Cr) 12 were different from other studies.

u‐EPX levels were higher in acute asthmatics when compared to chronic asthmatics reported by Gravesande et al. (estimated mean 233.3 ± SD 174.5 vs. estimated mean 124.7 ± SD 84.6 μg/mmol Cr) 25 and Oymar (estimated mean 134.67 ± SD 87.41 vs. estimated mean 96 ± SD 76.3 μg/mmol Cr 35. u‐EPX was higher in symptomatic asthmatic compared to asymptomatic asthmatic children 26, 28, 29, independent of treatment or atopy 28, 29. Similarly, elevated levels were seen in persistent atopic asthmatics versus persistent non‐atopic asthmatics 33. Labbe et al. and Lugosi et al. 27, 29 demonstrated that the levels of u‐EPX are independent of the atopic status and are higher among asthmatics than non‐asthmatics.

Eight studies did not support u‐EPX as a marker of inflammation in childhood asthma for estimating the course of disease 11, 22, 23, 31, 32, 34, 38, 39.

Treatment protocols affected urinary EPX levels differently in studies. The change in level of u‐EPX with medications (anti‐inflammatory) for asthma was demonstrated in nine studies 10, 20, 25, 27, 28, 29, 30, 32, 37, but a clear pattern could not be established. For instance, in a study by Nuijsink et al. 31, u‐EPX levels among asthmatics receiving different doses of fluticasone (200 and 500 μg/day) demonstrated varying u‐EPX levels of Median 189 (Range 2–2828) and median 180 (range 10*–*3114) μg/mmol. This wide range of U‐EPX levels makes it difficult to interpret the effect of different fluticasone doses on the u‐EPX levels. Nuijsink's studies 31, 32 used wheezing during the first year of life as an endpoint and determined that u‐EPX was not predictive for recurrent wheezing. Gravesande et al. 25 acute asthmatic group on 3 months of treatment with budesonide (400–600 μg) had similar u‐EPX levels (mean 75.8 ± SD 59.5 μg/mmol) as two other studies: (1) Kristjansson's acute asthmatic group on 3 months of budesonide and β_2_ agonists treatment (mean 68.4, 95%CI 41.4–95.4) 10, and (2) Kalaajieh's acute asthmatic group on 2 weeks of salbutamol and salmedarol (mean 66.5 ± SD 9.3) 26.

A total of nine studies used serum EPX levels as a measure of airway inflammation in asthma 4, 10, 11, 12, 17, 18, 19, 20, 21. Rao et al. 11 did not report any numerical results and Kim 17 compared mild, moderate, and severe asthmatics but with no comparison group. Remes's asthmatic group (estimated mean 64.23 μg/mmol) 19 was fairly similar to Zimmerman's atopic asthmatic group (Mean 69 μg/mmol) 20. Among the healthy control group, the central measures of serum EPX are comparable in the studies by Remes (estimated mean = 28.5 μg/mmol) 19, Kim (estimated mean = 24.03 μg/mmol) 18, Koller (estimated mean = 25.26 μg/mmol) 12, Kristjansson (estimated mean = 30.8 μg/mmol) 10, and Zimmerman (mean = 31.2 μg/mmol) 20.

Based on the qualitative results, we determined preliminary u‐EPX cut‐points for asthmatics and healthy controls as 134–140 μg/mmol for acute asthmatics; 65–75 μg/mmol Cr for acute asthmatics after treatment; and 56–61 μg/mmol Cr for healthy controls (based on five studies). However, these data were extremely limited and consisted of medians and quartiles, rather than means and standard deviations. Therefore, estimates of means (and SD) were calculated from reported medians (and quartiles).

### Quantitative analysis: diagnosing asthma with EPX

Due to the difficulty of diagnosing pediatric asthma in young children, several studies used terms such as airway inflammation, wheezing, or upper respiratory infections to describe asthmatics, making it difficult to determine if there were consistent trends of u‐EPX across these studies. Nine studies 25, 26, 27, 28, 29, 30, 33, 35, 40 reporting the levels of u‐EPX in uniformly defined asthmatic subgroups (i.e., acute asthmatics, asymptomatic asthmatics) and healthy controls were selected for meta‐analysis. Seven 25, 26, 27, 28, 29, 33, 35 out of nine studies reported u‐EPX levels for acute asthmatics and healthy controls, and five 26, 28, 29, 30, 40 out of nine studies reported u‐EPX levels for asymptomatic asthmatics and healthy controls with three studies 26, 28, 29 reporting u‐EPX levels for acute asthmatics, asymptomatic asthmatics, and healthy controls. One study, Severien et al. 37, which met the inclusion criteria for the quantitative analysis was not included in the meta‐analysis because of a high mismatch between reported (in the article) and the estimated standard deviations.

**Figure 2:**
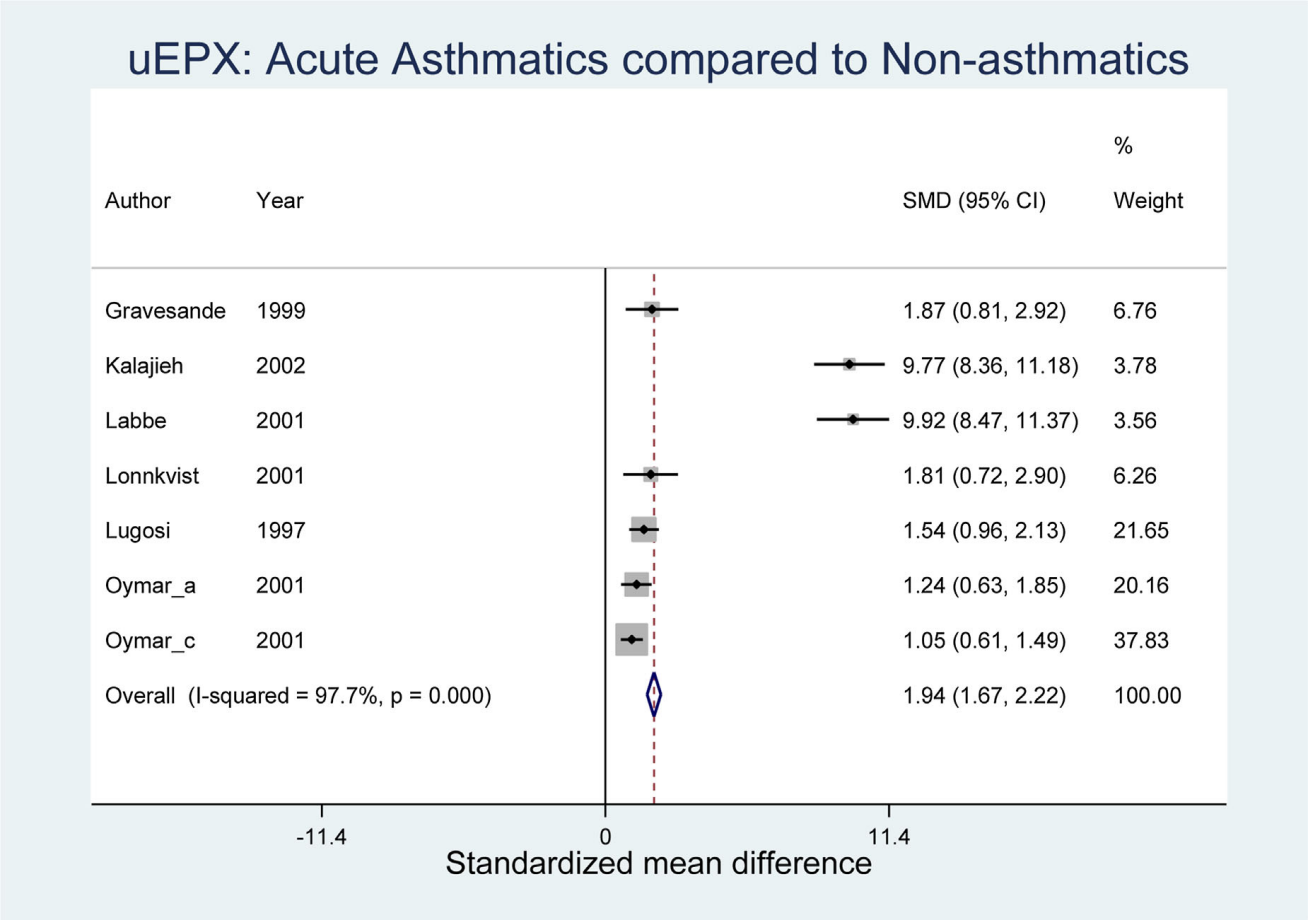
Forest plot showing standardized mean difference in urine eosinophil protein X (u‐EPX) levels among acute asthmatics and healthy controls.

**Figure 3:**
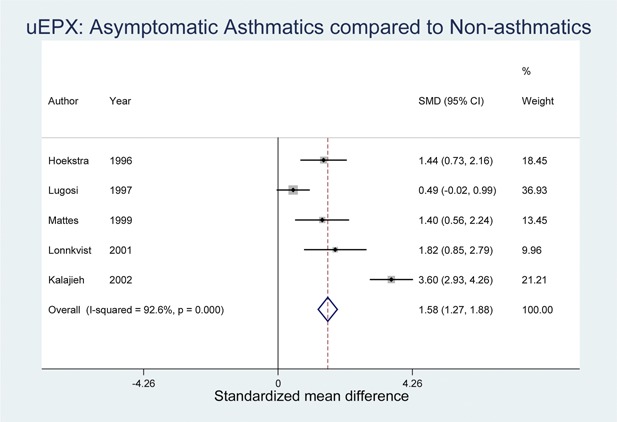
Forest plot showing standardized mean difference in urine eosinophil protein X (u‐EPX) levels among asymptomatic asthmatics and healthy controls.

The comparison of SMD between acute asthmatics and healthy controls, and asymptomatic (stable) asthma patients and healthy controls were performed and illustrated in Figures 2 and 3. Both forest plots indicate that net u‐EPX levels among asthmatics (irrespective of symptoms) are higher than those of non‐asthmatics. Comparing acute asthmatics and controls, the forest plot in Figure 2 suggests that, on average, the mean u‐EPX level among asthmatics is 1.94 times higher than those in the healthy control group with a confidence interval of 1.67–2.22. Similarly, the difference in standardized means between asymptomatic asthmatics and controls (Fig. 3) showed 1.58 times higher levels among asymptomatics than in healthy controls (CI: 1.27–1.88).

### Study quality assessment

The results of study quality assessment for the nine studies included in the quantitative analysis are reported in Table 5.

**Table 5:** Evaluation of quality of studies included in the meta‐analysis

	Research question	Study population	Selection bias	Inclusion/exclusion criteria	Measurement of exposure	Index test	Outcome measures
Gravesande (1999) 25	✓	✓	✓	✓	✓	✓	✓
Hoekstra (1996) 40	✓	✓	✓	✓	✓	✓	✓
Kalaajieh (2002) 26	✓	✓	✓	✓	✓	✓	✓
Labbe (2001) 27	✓	✓	✓	✓	✓	✓	✓
Lonnkvist (2001) 28	✓	X	✓	✓	✓	✓	X
Lugosi (1997) 29	✓	X	X	✓	✓	✓	✓
Mattes (1999) 30	✓	✓	✓	✓	✓	✓	✓
Oymar (2001) 35	✓	X	✓	✓	✓	✓	X
Oymar (2001) 33	✓	✓	✓	✓	✓	✓	✓

## Conclusions

There are currently a total of 27 studies investigating the role of urinary and serum EPX in pediatric asthma. Despite differences in study design, study sample size and characteristics (e.g., age or sex‐related differences), presence or absence of a comparison group, choice of accompanying laboratory (e.g., skin tests) and pulmonary tests, time of measurement (number of weeks prior to an exacerbation, pre‐ and/or post‐measurement), diagnostic criteria for asthma (e.g., based on history of recurrent varying number of episodes of wheezing [mild 1–2, moderate 3–12, and severe >12] within the past 12 months, and a physical exam by a physician), and varying endpoints, 19 out of 27 studies 10, 12, 17, 18, 19, 20, 21, 24, 25, 26, 27, 28, 29, 30, 33, 35, 36, 37, 40 reported a statistically significant relationships between EPX and childhood asthma.

A meta‐analysis was performed that reported u‐EPX values for both asthmatic cases and healthy controls. From the meta‐analysis of seven studies 25, 26, 27, 28, 29, 33, 35, it was determined that the overall SMD between acute asthmatics and non‐asthmatic healthy controls was 1.94 (CI: 1.67–2.22). Additionally, the meta‐analysis results of five studies 26, 28, 29, 30, 40 revealed an overall SMD of 1.58 (CI: 1.27–1.88) between asymptomatic asthmatic cases and healthy controls. Hence, Urinary EPX is elevated in children with either symptomatic or asymptomatic asthma compared to controls.

We attempted to establish preliminary u‐EPX cut‐points. The estimated cut‐points for acute asthmatics after treatment range from 65 to 75 µg/mmol Cr 10, 25, 26, with large standard deviations. For healthy controls, based on five studies, the u‐EPX range was from 56 to 61 µg/mmol Cr 27, 33, 35, 37, 40. However, there were three additional studies with ranges from 35 to 43 µg/mmol Cr 26, 27, 29. There were three studies that had extremely close results for acute asthmatics—from 134 to 140 µg/mmol Cr 26, 27, 35. However, there were four studies that did not reflect these values and had huge ranges and standard deviations: 103–233 µg/mmol Cr 25, 27, 28, 33. Hence, these cut points are not definitive, but serve as a first step towards further research in the diagnosis of asthma over time. Translating the proposed EPX cut‐points and values for diagnosing pediatric asthma into the clinical arena is not appropriate at this time. Accurate determination of cut points requires obtaining sensitivity and specificity measures for every study. At this time, there is no data in existence to assess sensitivity and specificity of u‐EPX measurement for diagnosis of pediatric asthma.

### Limitations

Although the present meta‐analysis establishes an association between u‐EPX and asthma and makes a case for clinical managing asthma, there remain a few limitations. Studies were conducted over 2 decades (from 1993 to 2015). This affected both the diagnostic criteria of childhood asthma as well as the methodologies used for estimating urine or serum EPX. Regardless, the diagnosis of asthma is exceedingly difficult to confirm in young children and infants, and usually requires years of follow‐up. Different criteria were used among the different studies (e.g., American Thoracic Society). Furthermore, the classification of asthma into mild, moderate, or severe was determined through a variety of clinical tests including asthma scores, chest X‐rays, measures of oxygen saturation, NO, and PEFR values.

Several studies were cross‐sectional and did not assess asthma over time. There were differences in methodologies including equipment and testing for EPX (which can significantly alter the values of u‐EPX and serum EPX) 41, and these measurements were not repeated post‐treatment. Circadian rhythm (lowest levels at 7 PM and highest at 7 AM) may have introduced scatter of u‐EPX 42, weakening possible correlations. There were also variations in performing PFTs (i.e., type, equipment) from one study to another.

Some studies included patients who were well‐controlled with treatment, thereby underestimating EPX levels during exacerbations. A few studies used comparison groups such as population‐based controls. There were also differences in sample selection, patient characteristics, and inclusion/exclusion criteria. Most studies were conducted in Europe on a small sample. All studies were based on whites, with no consideration of other racial/ethnic backgrounds.

Although u‐EPX is affected by age, symptomatic versus asymptomatic asthma, atopy, inhaled steroids, and infections specific for bronchial asthma 18, none of the studies adjusted for these effects. Additional confounding factors, including emotional triggers and environmental tobacco smoke, were also never considered.

### Future directions

To date, this is the first systematic review to implicate a useful role for urine EPX in childhood asthma, as well as suggest age‐specific cut‐points for urine EPX for asthmatics and healthy young children. Urinary EPX, a simple and non‐invasive technique, could be invaluable for diagnosing future asthma in children. Despite the limitations of the studies, a consistent trend of higher EPX levels with asthma was revealed. These results are too premature to draw any firm or generalizable conclusions. The utility of u‐EPX as a clinically relevant diagnostic metric in childhood asthma has yet to be established. Further research is required to replicate these findings and to validate this biomarker in a methodologically sound population‐based prospective cohort study consisting of measuring u‐EPX over several years in age‐specific (e.g., 0–4, 5–11, >12 years 43) children with newly diagnosed probable persistent asthma, stratified by severity (i.e., mild, moderate, and severe), prior to and after initiation of long‐term medications as well as during exacerbations, until a definitive diagnosis of pediatric asthma is confirmed. This will delineate the relationship between u‐EPX and the progression of asthma; thereby determining whether this biomarker is potentially useful in the clinical arena.
